# Clinical outcome of patients with recurrent or refractory localized Ewing's sarcoma family of tumors: A retrospective report from the Japan Ewing Sarcoma Study Group

**DOI:** 10.1002/cnr2.1329

**Published:** 2021-01-16

**Authors:** Katsutsugu Umeda, Takako Miyamura, Kenji Yamada, Hideki Sano, Ako Hosono, Minako Sumi, Hajime Okita, Tadashi Kumamoto, Akira Kawai, Junya Hirayama, Ryoji Jyoko, Akihisa Sawada, Hideki Nakayama, Yosuke Hosoya, Naoko Maeda, Nobuyuki Yamamoto, Chihaya Imai, Daiichiro Hasegawa, Motoaki Chin, Toshifumi Ozaki, Japan Ewing Sarcoma Study Group

**Affiliations:** ^1^ Department of Pediatrics, Graduate School of Medicine Kyoto University Kyoto Japan; ^2^ Department of Pediatrics Osaka University Graduate School of Medicine Suita Japan; ^3^ Department of Orthopedic Surgery Okazaki City Hospital Okazaki Japan; ^4^ Department of Pediatric Oncology National Cancer Center Hospital East Kashiwa Japan; ^5^ Department of Pediatric Oncology Fukushima Medical University Fukushima Japan; ^6^ Department of Radiation Oncology Tokyo Metropolitan Geriatric Hospital Tokyo Japan; ^7^ Department of Pathology Keio University School of Medicine Tokyo Japan; ^8^ Department of Pediatric Oncology National Cancer Center Hospital Tokyo Japan; ^9^ Musculoskeletal Oncology National Cancer Center Hospital Tokyo Japan; ^10^ Department of Pediatrics Mie University Graduate School of Medicine Tsu Japan; ^11^ Department of Orthopedic Surgery Okayama University Graduate School of Medicine, Dentistry and Pharmaceutical Sciences Okayama Japan; ^12^ Department of Hematology/Oncology Osaka Women's and Children's Hospital Izumi Japan; ^13^ Department of Pediatrics National Hospital Organization, Kyusyu Cancer Center Fukuoka Japan; ^14^ Department of Pediatrics St. Luke's International Hospital Tokyo Japan; ^15^ Department of Pediatrics National Hospital Organization Nagoya Medical Center Nagoya Japan; ^16^ Department of Pediatrics Kobe University Graduate School of Medicine Kobe Japan; ^17^ Department of Pediatrics Niigata University Graduate School of Medicine and Dental Sciences Niigata Japan; ^18^ Department of Hematology and Oncology Children's Cancer Center, Kobe Children's Hospital Kobe Japan; ^19^ Department of Pediatrics and Child Health Nihon University Itabashi Hospital Tokyo Japan

**Keywords:** chemotherapy, Ewing's sarcoma family of tumors, progression, relapse, stem cell transplantation

## Abstract

**Background:**

Patients with Ewing's sarcoma family of tumors (ESFT) who experience relapse or progression have a poor prognosis.

**Aim:**

This study aimed to identify the prognostic and therapeutic factors affecting overall survival (OS) of patients with recurrent or refractory localized ESFT.

**Methods and results:**

Thirty‐eight patients with localized ESFT who experienced first relapse or progression between 2000 and 2018 were retrospectively reviewed. The 5‐year OS rate of the entire cohort was 48.3% (95% confidence interval, 29.9%‐64.5%). Multivariate analysis of OS identified time to relapse or progression, but not stem cell transplantation (SCT), as the sole independent risk factor (hazard ratio, 35.8; *P* = .002). Among 31 patients who received salvage chemotherapy before local treatment, 21 received chemotherapy regimens that are not conventionally used for newly diagnosed ESFT. The objective response rate to first‐line salvage chemotherapy was 55.2% in the 29 evaluable patients. Time to relapse or progression was significantly associated with response to first‐line salvage chemotherapy (*P* = .006).

**Conclusions:**

The present study fails to demonstrate significant clinical benefit of SCT for recurrent or refractory localized ESFT. Recently established chemotherapy regimens may increase the survival rate of patients with recurrent or refractory localized ESFT while attenuating the beneficial effect of SCT.

**Abbreviations**
ACT: Actinomycin D
CBDCA: Carboplatin
CI: Confidence interval
CPA: Cyclophosphamide
CR: Complete response
CT: Computed tomography
DOC: Docetaxel
DXR: Doxorubicin
ESFT: Ewing's sarcoma family of tumors
ETP: Etoposide
EWSR: Ewing sarcoma breakpoint region
FISH: Fluorescent in situ hybridization
GEM: Gemcitabine
ICE: Ifosfamide+carboplatin+etoposide
IE: Ifosfamide+etoposide
IFM: Ifosfamide
IRI: Irinotecan
MEL: Melphalan
OS: Overall survival
PD: Progressive disease
PR: Partial response
RFI: Relapse‐free interval
SCT: Stem cell transplantation
SD: Stable disease
TMZ: Temozolomide
TPT: Topotecan
VCR: Vincristine
VDC: Vincristine+doxorubicin+cyclophosphamide

## 1 INTRODUCTION

Ewing's sarcoma family of tumors (ESFT), the second most frequent bone tumor in children and young adults, is genetically characterized by a fusion involving Ewing sarcoma breakpoint region (EWSR)1 gene and a member of the ETS family of transcription factors.^1^ Introduction of first‐line multidrug regimens, consisting of three to six combination of key drugs [vincristine (VCR), doxorubicin (DXR), cyclophosphamide (CPA), ifosfamide (IFM), etoposide (ETP), and actinomycin D (ACT)], led to markedly improved outcome of patients with ESFT. Consequently, intensified multidrug chemotherapy, achieved by increasing the dose or by decreasing the interval between chemotherapy cycles, in combination with surgery and radiotherapy has contributed to a high survival rate of 70% to 80%. However, approximately 30% to 40% of patients with localized ESFT experience relapse or progression.^1^, ^2^, ^3^, ^4^, ^5^, ^6^, ^7^ The prognosis of these patients is extremely poor, with a long‐term survival rate of approximately 10% to 30%.^8^, ^9^, ^10^, ^11^, ^12^, ^13^ Time to relapse or progression,^8^, ^10^, ^12^, ^13^ type of relapse or progression,^11^, ^12^ response to first‐line salvage chemotherapy,^13^ and stem cell transplantation (SCT) are strong prognostic factors for overall survival (OS).^13^

Because standard salvage chemotherapy has not been established to date, another combination of these key drugs was previously used as salvage chemotherapy for recurrent or refractory ESFT, in combination with local treatment (surgery and radiotherapy) for the primary site and/or metastasis. However, recurrent or refractory ESFT is often resistant to these chemotherapy regimens. Recently established chemotherapy regimens, including IFM + VP16 + carboplatin (CBDCA, ICE), topotecan (TPT) + CPA, temozolomide (TMZ) + irinotecan (IRI), and gemcitabine (GEM) + docetaxel (DOC), are effective in a fraction of patients with recurrent or refractory ESFT.^14^, ^15^, ^16^, ^17^, ^18^ These regimens may change the clinical impact of prognostic factors over time. In the current study, we retrospectively analyzed the clinical outcomes of patients with localized ESFT who experienced relapse or progression to evaluate the prognostic factors affecting OS in the recent era.

## 2 MATERIALS AND METHODS

### 2.1 Study design and data collection

This study was approved by the Clinical Research Review Committee of the Japan Children's Cancer Group and the Institutional Ethics Committee of Kyoto University Hospital. Data from 47 patients with localized ESFT who experienced first relapse or progression between 2000 and 2018 were obtained from 30 institutions by self‐administered questionnaire. Of these, nine patients were excluded due to a lack of data on survival status (n = 2) or EWS‐ETS fusion gene (n = 6). One patient who did not relapse was also excluded. EWS‐ETS fusion genes, including *EWS‐FLI1* (n = 23), *EWS‐ERG* (n = 1), and *EWS‐FEV* (n = 2), were detected in 26 patients by reverse transcription polymerase chain reaction. In the remaining 12 patients, the EWSR1 translocation was detected by fluorescent in situ hybridization (FISH) using break‐apart probes. In total, 38 patients who were diagnosed as ESFT by molecular testing were analyzed.

Relapse or progression was confirmed by imaging including computed tomography (CT), magnetic resonance imaging, or positron emission tomography‐CT in all patients. Histological analyses were not routinely performed to confirm relapse or progression. Relapse‐free interval (RFI) was defined as the time from initial diagnosis to first relapse, as previously reported.^13^ A cut‐off RFI value of 2 years was set according to the previous report.^13^ Radiological response to chemotherapy was evaluated according to the RECIST guidelines (version 1.1).^19^

### 2.2 Statistical analysis

The probability of OS, defined as the duration of survival between first relapse or progression and either death or the last follow‐up, was estimated using the Kaplan–Meier method; the log‐rank test and the Cox proportional hazard model were used for univariate and multivariate analyses, respectively. The factors included in the analyses were patient age group (0‐12 years vs ≥13), gender (male vs female), fusion gene (*EWS‐FLI1* vs *EWS‐ERG* vs *EWS‐FEV* vs EWSR1‐FISH), primary tumor origin (bone vs soft tissue), primary tumor site (extremity vs axial vs other), time to relapse or progression (off therapy vs on therapy), relapse‐free interval (≥2 years vs <2 years), type of relapse or progression [local vs metastatic vs combined (local and synchronal metastatic)], response to first‐line salvage chemotherapy [complete response (CR)/partial response (PR) vs stable disease (SD)/progressive disease (PD)], and SCT for relapse or progression (no vs yes). Factors with *P* < .1 in the univariate analysis were included in the multivariate analysis. Pearson's chi‐squared test and a logistic regression model were used for univariate analysis of response to first‐line salvage chemotherapy. All statistical analyses were performed using EZR (version 1.32, Saitama Medical Center, Jichi Medical University), which is a graphical user interface for R (the R Foundation for Statistical Computing).^20^

## 3 RESULTS

### 3.1 Patient characteristics at initial diagnosis and treatment

Of 50 surveyed institutions, 30 (60.0%) responded. The characteristics at initial diagnosis and the treatment of the 38 patients included in the study are presented in Table 1. The median age at initial diagnosis was 13 years (range, 1‐29 years). Among 31 patients receiving initial chemotherapy before local treatment, 24 received VCR + DXR + CPA (VDC)/IFM + ETP (IE) at 2‐week (n = 9) or 3‐week (n = 15) intervals. Of the 29 patients evaluable for radiological response to initial chemotherapy before local treatment, there were 3 CR, 19 PR, 4 SD, and 3 PD, with an objective response rate (CR + PR) of 75.9%. The remaining seven patients who initially underwent surgery at the primary site received postoperative chemotherapy, including VDC/IE (n = 4), VCR + ACT+IFM + DXR (n = 1), and others (n = 2). Four patients underwent SCT during initial treatment.

**TABLE 1:** Patient characteristics at initial diagnosis and treatment

Characteristics	All patients (n = 38)	
	No.	%
Sex		
Male	22	57.9
Female	16	42.1
Age at diagnosis (years)		
Median (range)	13.5 (1–29)	
Primary tumor site		
Extremity	19	50.0
Axial	13	34.2
Other	6	15.8
Primary tumor origin		
Bone	25	65.8
Soft tissue	13	34.2
Primary tumor volume (mL)		
Median (range)	197 (15‐1893)	
<200 mL	13	34.2
≥200 mL	13	34.2
Missing	12	31.6
Fusion gene		
EWS‐FLI1	23	60.5
EWS‐ERG	1	2.6
EWS‐FEV	2	5.3
EWSR1‐FISH	12	31.6
Initial chemotherapy before local treatment		
VDC/IE q2w	9	23.7
VDC/IE q3w	15	39.5
VAIA	3	7.9
VIDE	2	5.3
Other	1	2.6
Missing	1	2.6
No	7	18.4
Local treatment for primary site		
Surgery	21	55.2
Radiotherapy	5	13.2
Surgery and radiotherapy	10	26.3
No	2	5.3
SCT before relapse or progression		
No	34	89.5
Yes	4	10.5

Abbreviations: EWSR, Ewing's sarcoma region; IE, ifosfamide+etoposide; q2w, every 2 weeks; q3w, every 3 weeks; SCT, stem cell transplantation; VAIA, vincristine+actinomycin+ifosfamide+doxorubicin; VDC, vincristine+doxorubicin+cyclophosphamideVIDE, vincristine+ifosfamide+doxorubicin+etoposide.

### 3.2 Patient characteristics at first relapse or progression

The clinical features of 38 patients at first relapse or progression are presented in Table 2. The median RFI was 8 months (range, 0‐46 months). Of the 38 patients, 30 (78.9%) experienced relapse or progression off therapy. Twenty‐six (68.4%) patients developed metastatic (without local) relapse or progression. Thirty‐one patients received first‐line salvage chemotherapy before local treatment, whereas the remaining seven patients initially underwent local treatment. Among the 31 patients who received chemotherapy before local treatment, 21 were treated with first‐line salvage regimens that are not conventionally used for newly diagnosed ESFT, including TPT‐based chemotherapy (n = 8), TMZ + IRI ± VCR (n = 6), ICE (n = 6), and GEM+DOC (n = 1). Among the seven patients initially receiving local treatment, four received first‐line salvage chemotherapy such as TMZ + IRI ± VCR (n = 2), TPT‐based chemotherapy (n = 1), and VDC/IE at 3‐week intervals (n = 1). The local treatment for the primary site was surgery in four patients, radiotherapy in three patients, and both treatments in one patient. The local treatment for metastasis was surgery in 7 patents, radiotherapy in 15 patients, and both treatments in 3 patients. Sixteen patients underwent SCT for first relapse or progression.

**TABLE 2:** Patient characteristics at first relapse or progression

Characteristics	All patients (n = 38)	
	No.	%
Relapse‐free interval (months)		
Median (range)	8 (0‐46)	
<24 months	26	68.4
≥24 months	12	31.6
Time of relapse		
On therapy	8	21.1
Off therapy	30	78.9
Type of relapse or progression		
Local	9	23.7
Metastatic	26	68.4
Combined	3	7.9
First‐line salvage chemotherapy before local treatment		
VDC/IE q3w	4	10.5
TPT‐based	8	21.1
TMZ/IRI ± VCR	6	15.8
ICE	6	15.8
GEM+DOC	1	2.6
Other	6	15.8
No	7	18.4
Local treatment for primary site		
Surgery	4	10.5
Radiotherapy	3	7.9
Surgery and radiotherapy	1	2.6
No	30	78.9
Local treatment for metastasis		
Surgery	7	18.4
Radiotherapy	15	39.5
Surgery and radiotherapy	3	7.9
No	13	34.2
SCT for relapse or progression		
No	22	57.9
Yes	16	42.1
Follow‐up period (months)		
Median (range)	19 (3‐133)	

Abbreviations: DOC, docetaxel; GEM, gemcitabine; ICE, ifosphamide+carboplatin+etoposide; IE, ifosfamide+etoposide; IRI, irinotecan; q3w, every 3 weeks; SCT, stem cell transplantation; TMZ, temozolimide; TPT, topotecan; VCR, vincristine; VDC, vincristine+doxorubicin+cyclophosphamide.

### 3.3 Factors affecting OS

The 5‐year OS rate of the entire cohort was 48.3% [95% confidence interval (CI), 29.9%‐64.5%]. Treatment‐related death was observed in three patients who underwent allogeneic SCT. No secondary malignancy was observed. In the univariate analysis, time to relapse or progression, type of relapse or progression, and response to first‐line salvage chemotherapy, but not SCT, were identified as risk factors for OS. Multivariate analysis identified time to relapse or progression as the sole independent risk factor for OS (adjusted hazard ratio 35.8; 95% CI, 3.54‐363.2, *P* = .002; Table 3).

**TABLE 3:** Univariate and multivariate analyses of factors affecting OS

Variables	Factors (n)	5 years OS, % (95% CI)	Univariate analysis	Multivariate analysis	
			*P*‐value	HR (95% CI)	*P*‐value
Age group	0‐12 (18)	61.5 (33.3‐80.7)	.144	N.E.	N.E.
	≥13 (20)	34.9 (12.5‐58.7)			
Gender	Male (22)	42.2 (19.4‐63.5)	.124	N.E.	N.E.
	Female (16)	56.1 (26.2‐77.9)			
Fusion gene	EWS‐FLI1 (23)	45.5 (22.7‐65.8)	.590	N.E.	N.E.
	EWS‐ERG (1)	100			
	EWS‐FEV (2)	0			
	EWS‐FISH (12)	52.9 (20.5‐77.4)			
Primary tumor origin	Bone (25)	52.5 (30.3‐70.6)	.329	N.E.	N.E.
	Soft tissue (13)	36.3 (8.8‐65.5)			
Primary tumor site	Extremity (13)	40.3 (13.7‐66.0)	.768	N.E.	N.E.
	Axial (19)	56.1 (27.4‐77.3)			
	Other (6)	50.0 (11.1‐80.4)			
Time of relapse	Off therapy (30)	61.8 (38.7‐78.3)	<.001	Reference	
	On therapy (8)	0		35.8 (3.54‐363.2)	.002
Relapse‐free interval	<24 months (26)	47.9 (25.5‐67.2)	.581	N.E.	N.E.
	≥24 months (12)	50.0 (18.4‐75.3)			
Type of relapse or progression	Local (9)	37.0 (1.4‐79.4)	<.001	Reference	
	Metastatic (26)	53.1 (31.2‐71.0)		0.83 (0.22‐3.12)	.779
	Combined (3)	0		3.06 (0.37‐25.5)	.301
Response to first‐line salvage chemotherapy	CR/PR (16)	53.1 (23.6‐75.7)	.026	Reference	
	SD/PD (13)	17.9 (1.3‐50.4)		2.04 (0.64‐6.51)	.229
SCT for relapse or progression	No (22)	53.4 (28.2‐73.3)	.610	N.E.	N.E.
	Yes (16)	42.5 (16.7‐66.4)			

Abbreviations: CI, confidence interval; CR, complete response; EWSR, Ewing's sarcoma region; FISH, fluorescent in situ hybridization; HR, hazard ratio; N.E., not evaluated; OS, overall survival; PD, progressive disease; PR, partial response; SCT, stem cell transplantation; SD, stable disease.

**FIGURE 1:**
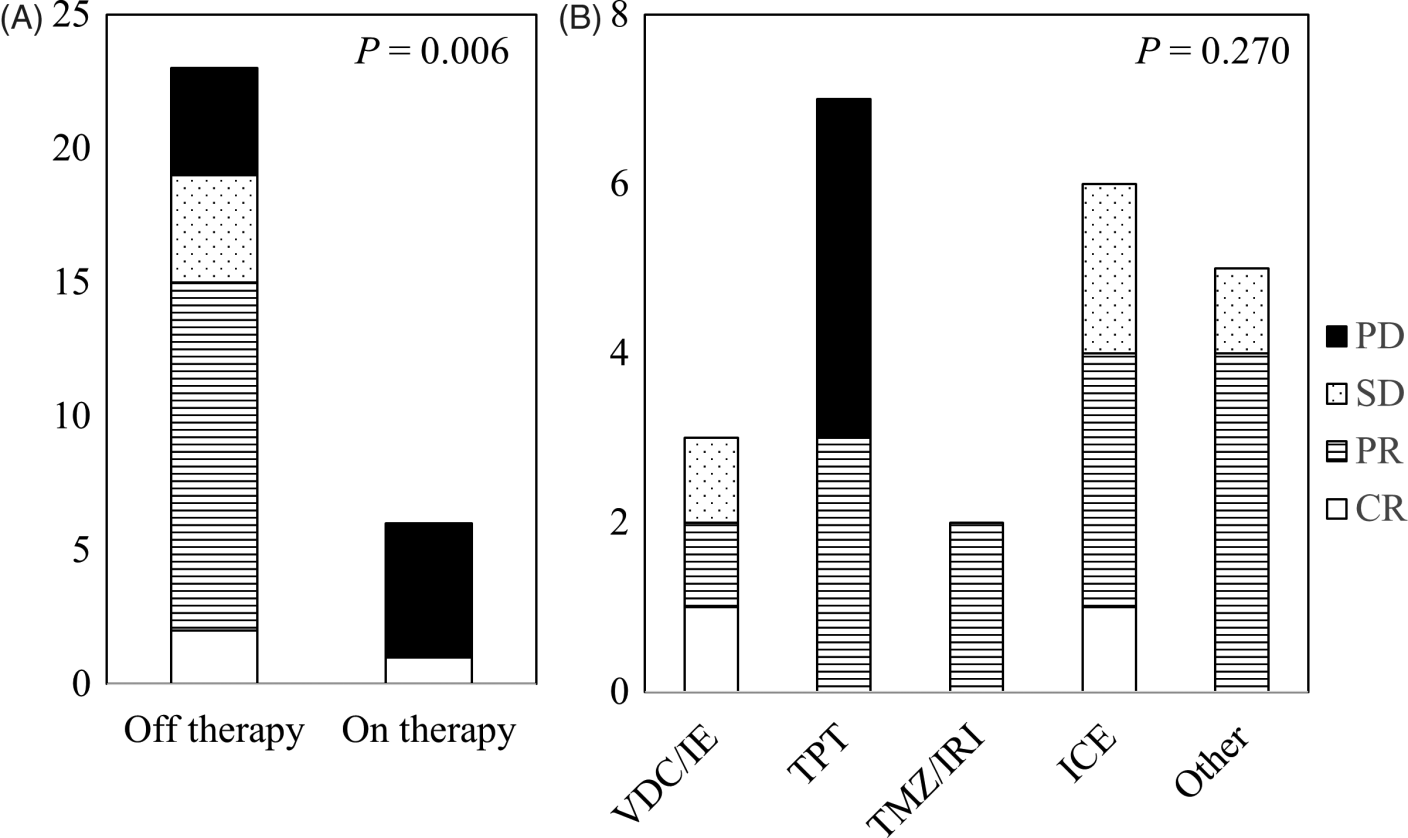
A, B, Radiological response to first‐line salvage chemotherapy before local treatment grouped by time to relapse or progression A, and type of salvage chemotherapy B. CR, complete response; PR, partial response; SD, stable disease; PD, progressive disease; VDC, vincristine+doxorubicin+cyclophosphamide; IE, ifosfamide+etoposide; TPT, topotecan; TMZ, temozolomide; IRI, irinotecan; ICE, ifosfamide+carboplatin+etoposide

Of the 29 patients evaluable for radiological response to first‐line salvage chemotherapy before local treatment, there were three CR, 13 PR, four SD, and nine PD, with an objective response rate of 55.2%. Of 23 patients experiencing relapse or progression off therapy, 15 achieved CR or PR, whereas one of six patients experiencing relapse or progression on therapy achieved CR or PR (*P* = .006, Figure 1A). Among the 23 patients experiencing relapse or progression off therapy, there was no significant difference in response according to the type of chemotherapy (*P* = .270, Figure 1B).

### 3.4 Impact of SCT on clinical outcome

The clinical information of 16 patients undergoing SCT for relapse or progression is presented in Table 4. Eleven patients received single autologous SCT, one received tandem autologous SCT, two received single allogeneic SCT, and two received tandem autologous‐allogeneic SCT. The most common conditioning regimens were busulfan+melphalan (MEL) (n = 9) and CBDCA+ETP + MEL (n = 3). The 5‐year OS rate of 16 patients who underwent SCT after relapse or progression (42.5%; 95% CI, 16.7%‐66.4%) was comparable to that of 22 patents who did not (53.4%; 95% CI, 28.2%‐73.3%; *P* = .610; Table 3). All four patients undergoing allogeneic SCT died of complications (n = 3) or disease progression (n = 1).

**TABLE 4:** Clinical information of patients undergoing SCT for relapse or progression

No.	Age at diagnosis (yr)	Sex	Type of relapse or progression	Disease status before SCT	First SCT source (regimen)	Second SCT source (regimen)	Outcome (mo)
1	8	F	Metastatic	SD	Auto‐PB (ETP)	‐	10 (DOD)
2	19	F	Metastatic	PR	Auto‐PB (BU, MEL)	MMR‐PB (FLU, MEL, ATG)	13 (DOC)
3	20	M	Metastatic	PR	MMR‐PB (FLU, MEL, ATG)	‐	18 (DOC)
4	16	F	Local	PD	Auto‐PB (IFO, CBDCA, ETP)	MR‐PB (FLU, BU)	18 (AWD)
5	4	M	Local	PD	UR‐CB (FLU, MEL, ETP)	‐	10 (DOC)
6	17	M	Local	PD	Auto‐PB (BU, MEL)	‐	4 (DOD)
7	17	M	Metastatic	PR	Auto‐PB (BU, MEL)	‐	42 (DOD)
8	10	F	Metastatic	CR	Auto‐PB (BU, MEL)	‐	22 (DOD)
9	12	F	Metastatic	PR	Auto‐PB (BU, MEL)	‐	9 (NED)
10	8	M	Metastatic	CR	Auto‐PB (CBDCA, ETP, MEL)	Auto‐PB (TBI, TEPA)	97 (DOD)
11	16	F	Metastatic	CR	Auto‐PB (BU, MEL)	‐	22 (NED)
12	14	F	Metastatic	PR	Auto‐PB (BU, MEL)	‐	60 (NED)
13	9	F	Metastatic	CR	Auto‐PB (CBDCA, ETP, MEL)	‐	81 (NED)
14	10	F	Metastatic	CR	Auto‐PB (BU, MEL)	‐	91 (NED)
15	24	M	Metastatic	CR	Auto‐PB (BU, MEL)	‐	61 (NED)
16	14	F	Metastatic	CR	Auto‐PB (CBDCA, ETP, MEL)	‐	9 (DOD)

Abbreviations: ATG, anti‐thymocyte globulin; Auto‐PB, autologous peripheral blood stem cells; AWD, alive with disease; BU, busulfan; CBDCA, carboplatin; CR, complete response; DOC, died of complications; DOD, died of disease; ETP, etoposide; F, female; FLU, fludarabine; IFO, ifosfamide; M, male; MEL, melphalan; MMR‐PB, HLA‐mismatched related peripheral blood stem cells; mo, months; MR‐PB, HLA‐matched related peripheral blood stem cells; NED, no evidence of disease; PD, progressive disease; PR, partial response; SCT, stem cell transplantation; SD, stable disease; TBI, total body irradiation; TEPA, thiotepa; UR‐CB, unrelated cord blood; yr, years.

**FIGURE 2:**
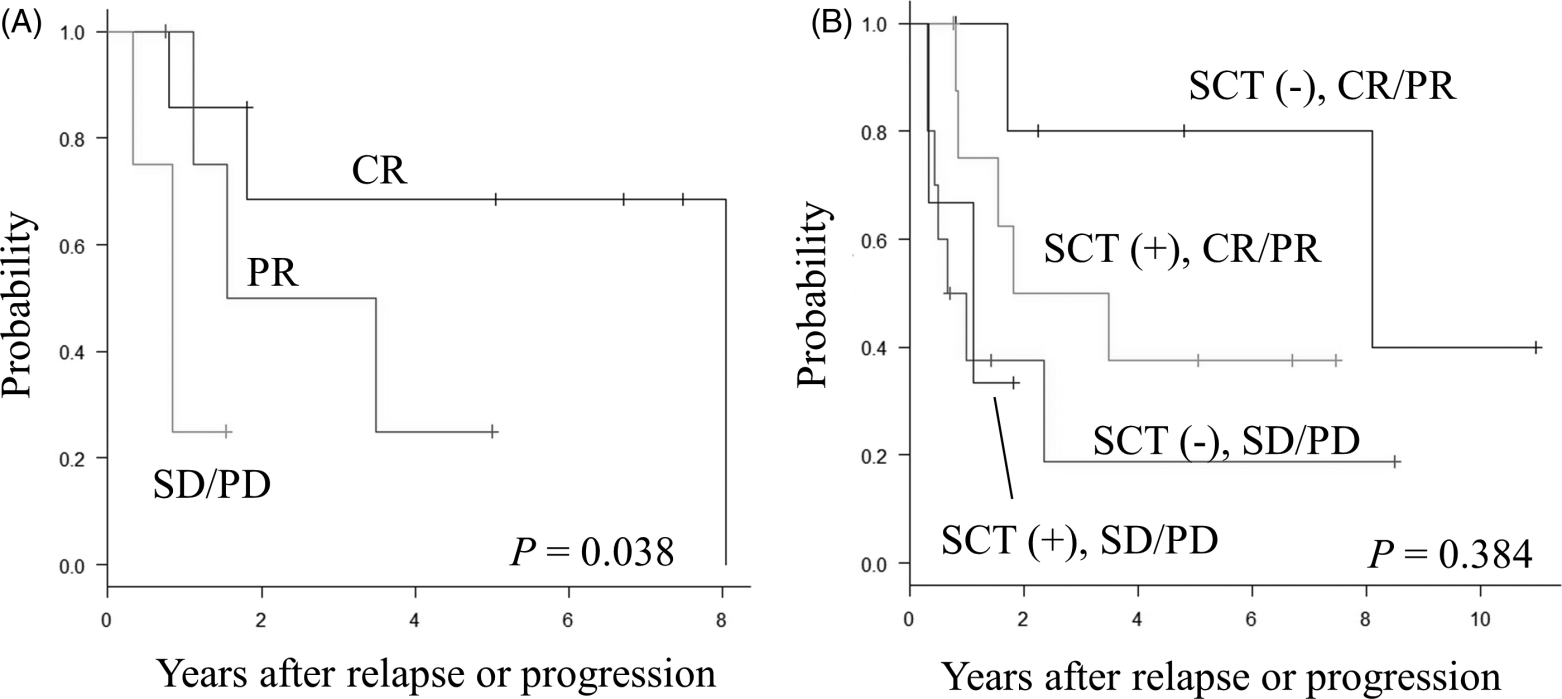
A, B OS rates of patients who underwent SCT grouped by disease status before SCT, A, and response to first‐line salvage chemotherapy and SCT, B. CR, complete response; PR, partial response; SD, stable disease; PD, progressive disease

Next, we analyzed the influence of other confounding factors on the significance of SCT. Among patients who underwent SCT for relapse or progression, the 5‐year OS rates grouped by disease status before SCT were 68.6% (95% CI, 21.3%‐91.2%) in seven patients with CR, 25.0% (95% CI, 0.9%‐66.5%) in five patients with PR, and 0% in four patients with SD/PD (*P* = .038, Figure 2A). The 5‐year OS rates grouped by response to first‐line salvage chemotherapy and SCT were 37.5% (95% CI, 8.7%‐67.4%) in nine patients with CR/PR who underwent SCT, 80.0% (95% CI, 20.4%‐96.9%) in seven patients with CR/PR who did not, 0% in three patients with SD/PD who underwent SCT, and 18.8% (95% CI, 1.2%‐52.9%) in 10 patients with SD/PD who did not (*P* = .384, Figure 2B).

### 3.5 Impact of local treatment on clinical outcome

To evaluate the impact of local treatment for recurrent or refractory ESFT, we analyzed the OS rates of patients with primary site or metastatic disease alone. Among nine patients with primary site involvement alone, the 5‐year OS rate was higher in five patients who underwent surgery (80.0%,; 95% CI, 20.4%‐96.9%) than in four patients who did not, although the difference was not statistically significant (0%, *P* = .384, Figure S1a). Among 26 patients with metastasis alone, the 5‐year OS rate was higher in eight patients who underwent surgery with or without radiotherapy (85.7%; 95% CI, 33.4%‐97.9%) than in 13 patients receiving radiotherapy alone (29.9%; 95% CI, 7.5%‐57.0%) or five patients without local treatment (60.0%; 95% CI, 12.6%‐88.2%), although the difference was not statistically significant (*P* = .196, Figure S1b).

## 4 DISCUSSION

The 5‐year OS rate in our patient cohort was 48.3%, which is relatively higher than that of patients with recurrent or refractory localized ESFT reported previously (20%‐30%).^8^, ^13^ The objective response rate to first‐line salvage chemotherapy (55.2%) was also higher than that reported previously.^9^, ^13^ In the present study, more than half of the patients received ICE, TPT‐based chemotherapy, and TMZ + IRI ± VCR, which are not conventionally used for newly diagnosed ESFT, whereas nearly half of the patients conventionally received IE in previous reports.^9^, ^13^ These observations suggest that recently established regimens may increase the response rate to chemotherapy and the survival rate of patients with recurrent or refractory localized ESFT. Another possible explanation for the difference in OS is that the present study included a higher proportion of younger patients with a better outcome, although age at initial diagnosis was not identified as an independent prognostic factor. Furthermore, selection bias potentially introduced by data from patients with a better clinical outcome may affect OS. Nonetheless, a prospective study including larger populations is required to establish the optimal treatment strategy incorporating novel chemotherapy regimens to increase the curative rate of recurrent or refractory ESFT.

Time to relapse or progression, type of relapse or progression, response to first‐line salvage chemotherapy, and SCT are strong prognostic factors for OS in patients with recurrent or refractory ESFT.^8^, ^10^, ^11^, ^12^, ^13^ In the present study, time to relapse or progression was significantly related to response to first‐line salvage chemotherapy and identified as the sole independent risk factor affecting OS. These observations suggest the limitations of currently available chemotherapy regimens for patients who experience relapse or progression on therapy. Because the efficacy of molecular‐targeted therapy, such as monoclonal antibody to the insulin‐like growth factor 1 receptor, is limited for a subgroup of patients with ESFT,^21^, ^22^ comprehensive molecular profiling for targeted therapy is a prerequisite for tailoring personalized therapies.

Regarding the type of relapse or progression, the outcome after combined relapse or progression is markedly inferior to that after local relapse or progression alone or metastatic relapse or progression alone.^11^, ^12^ In the present study, the three patients with combined relapse or progression died from the disease; however, the type of relapse or progression was not identified as an independent prognostic factor probably because of the paucity of available data.

High‐dose chemotherapy with SCT is used for recurrent or refractory ESFT to overcome relative resistance to chemotherapy. However, the clinical significance of SCT remains controversial. Several analyses were unable to address the clinical significance of SCT because few patients achieved CR or PR after salvage chemotherapy and underwent SCT.^23^, ^24^ Furthermore, recent reports demonstrating the clinical efficacy of SCT exclude patients with chemotherapy‐resistant disease who could not achieve CR or PR, which introduces selection bias favoring patients following a better clinical course or receiving treatment in highly specialized hospitals.^13^, ^25^ The present study included patients with SD or PD after salvage chemotherapy and confirmed the clinical significance of disease status before SCT. However, we found that response to first‐line salvage chemotherapy affects the clinical outcome irrespective of SCT. The clinical significance of conditioning regimens, tandem HSCT, or allogeneic HSCT was not evaluated because of the low number of patients included in this study. Nonetheless, the present study fails to demonstrate significant clinical benefit of SCT for recurrent or refractory localized ESFT.

Previous reports demonstrate that the OS rate of patients who receive local therapy for primary tumors or metastatic disease is significantly higher than that of patients who do not.^8^, ^10^ The present study showed a similar trend: however, significant differences were not observed because of the paucity of available data. Furthermore, the data should be interpreted with caution because patients with an expected poor prognosis associated with the contraindication to local treatment were treated with chemotherapy alone.

The present study had several limitations. First, it was a retrospective analysis of data from a heterogeneous group of patients, which hampered the statistical evaluation of certain prognostic and therapeutic factors affecting clinical outcome. Second, we did not examine the histological response to chemotherapy because of a lack of data in most patients. Lastly, the follow‐up period was too short to evaluate late adverse effects, such as secondary malignancies and infertility. Nonetheless, the relatively large cohort of patients with rare recurrent or refractory localized ESFT in the present study led to the important observation that recently established chemotherapy regimens may increase the survival rate of recurrent or refractory localized ESFT without SCT. Further prospective study is required to establish personalized targeted therapies for patients experiencing relapse or progression on therapy with extremely poor prognosis.

## CONFLICT OF INTEREST

The authors have no conflicts of interest to declare.

## AUTHOR CONTRIBUTIONS

All authors had full access to the data in the study and take responsibility for the integrity of the data and the accuracy of the data analysis. *Conceptualization*, K.U., T.M., K.Y., H.S., M.S., H.O., M.C., T.O.; *Methodology*, K.Y.; *Investigation*, K.U., T.M., K.Y., T.K., A.H., J.H., R.J., A.S., H.N., Y.H., N.M., N.Y., C.I., D.H., T.O.; *Formal Analysis*, K.U., T.M., A.H., M.S., H.O.; *Writing ‐ Original Draft*, K.U., T.M.; *Writing ‐ Review & Editing*, K.U., H.S., A.H., M.S., H.O., M.C., T.O.; *Visualization*, K.U.; *Supervision*, H.S., A.H., M.S., H.O., M.C., T.O., *Validation*, K.Y.; *Project Administration*, H.S., A.H., M.S., H.O., T.O.

## ETHICAL STATEMENT

All procedures performed in studies involving human participants were in accordance with the ethical standards of the institutional research committee and with the 1964 Helsinki Declaration and its later amendments or comparable ethical standards. This retrospective study was approved by the institutional review board, and the requirement to obtain informed consent was waived.

## Supporting information


**Figure S1.** (a) OS rates of patients who experienced relapse or progression of primary site alone grouped by surgery. (b) OS rates of patients with metastasis alone grouped by local treatment.

## Data Availability

The data are not publicly available because of privacy or ethical restriction.
