# Supplementary figures and images for: Clinical outcome of patients with recurrent or refractory localized Ewing's sarcoma family of tumors: A retrospective report from the Japan Ewing Sarcoma Study Group
Source: Cancer Rep (Hoboken). 2021 Jan 16;4(3):e1329. doi: 10.1002/cnr2.1329 (PMC8222563; doi:10.1002/cnr2.1329)

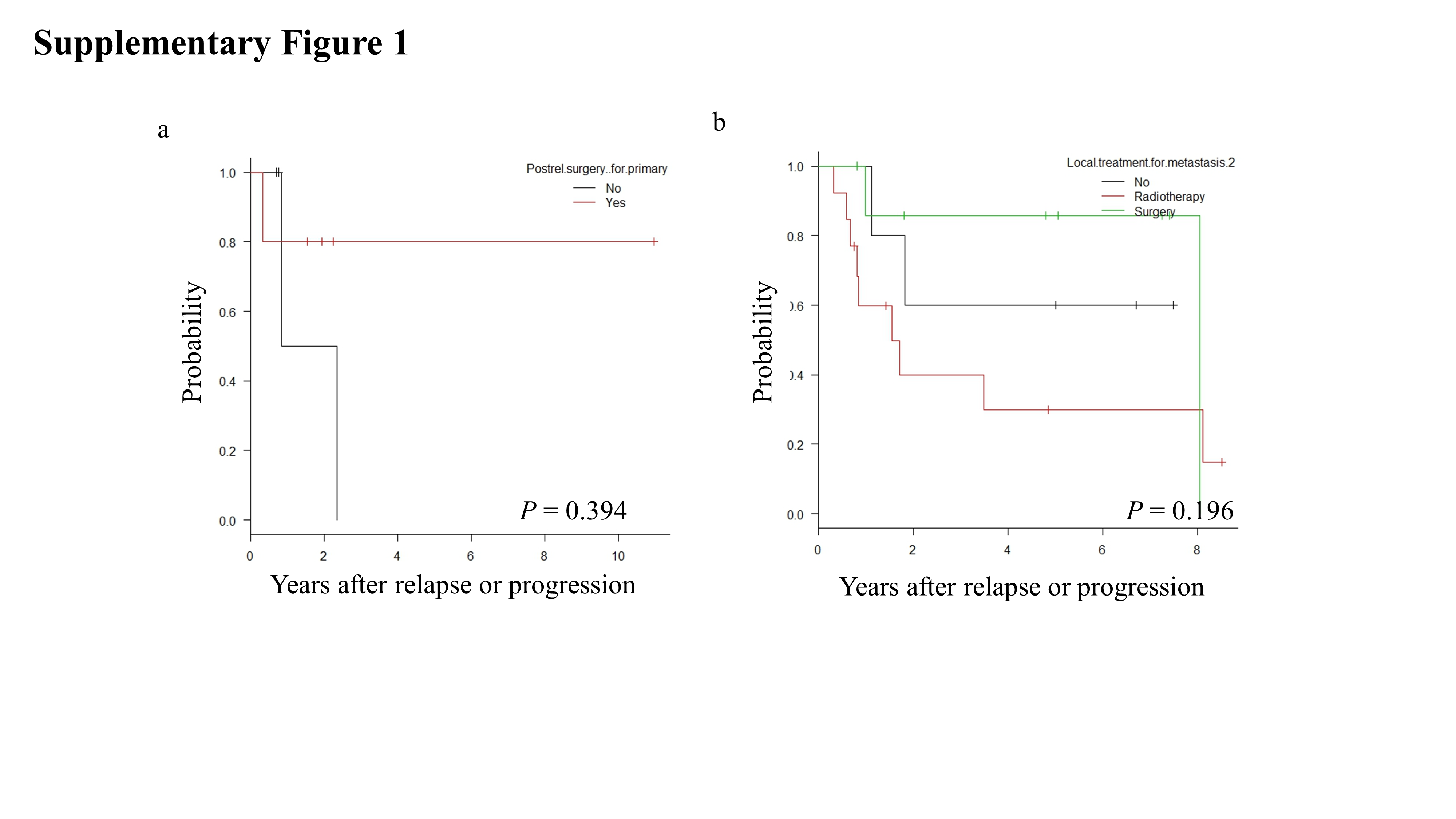

Supplement: Supplementary file 1 — Figure S1. (a) OS rates of patients who experienced relapse or progression of primary site alone grouped by surgery. (b) OS rates of patients with metastasis alone grouped by local treatment. [file CNR2-4-e1329-s001.tif]
